# On the Developmental and Environmental Regulation of Secondary Metabolism in *Vaccinium* spp. Berries

**DOI:** 10.3389/fpls.2016.00655

**Published:** 2016-05-18

**Authors:** Katja Karppinen, Laura Zoratti, Nga Nguyenquynh, Hely Häggman, Laura Jaakola

**Affiliations:** ^1^Genetics and Physiology Unit, University of Oulu, OuluFinland; ^2^Climate laboratory Holt, Department of Arctic and Marine Biology, UiT the Arctic University of Norway, TromsøNorway; ^3^NIBIO, Norwegian Institute of Bioeconomy Research, ÅsNorway

**Keywords:** anthocyanins, bilberry, blueberry, carotenoids, flavonoids, fruits, light, temperature

## Abstract

Secondary metabolites have important defense and signaling roles, and they contribute to the overall quality of developing and ripening fruits. Blueberries, bilberries, cranberries, and other *Vaccinium* berries are fleshy berry fruits recognized for the high levels of bioactive compounds, especially anthocyanin pigments. Besides anthocyanins and other products of the phenylpropanoid and flavonoid pathways, these berries also contain other metabolites of interest, such as carotenoid derivatives, vitamins and flavor compounds. Recently, new information has been achieved on the mechanisms related with developmental, environmental, and genetic factors involved in the regulation of secondary metabolism in *Vaccinium* fruits. Especially light conditions and temperature are demonstrated to have a prominent role on the composition of phenolic compounds. The present review focuses on the studies on mechanisms associated with the regulation of key secondary metabolites, mainly phenolic compounds, in *Vaccinium* berries. The advances in the research concerning biosynthesis of phenolic compounds in *Vaccinium* species, including specific studies with mutant genotypes in addition to controlled and field experiments on the genotype × environment (G×E) interaction, are discussed. The recently published *Vaccinium* transcriptome and genome databases provide new tools for the studies on the metabolic routes.

## Introduction

**FIGURE 1:**
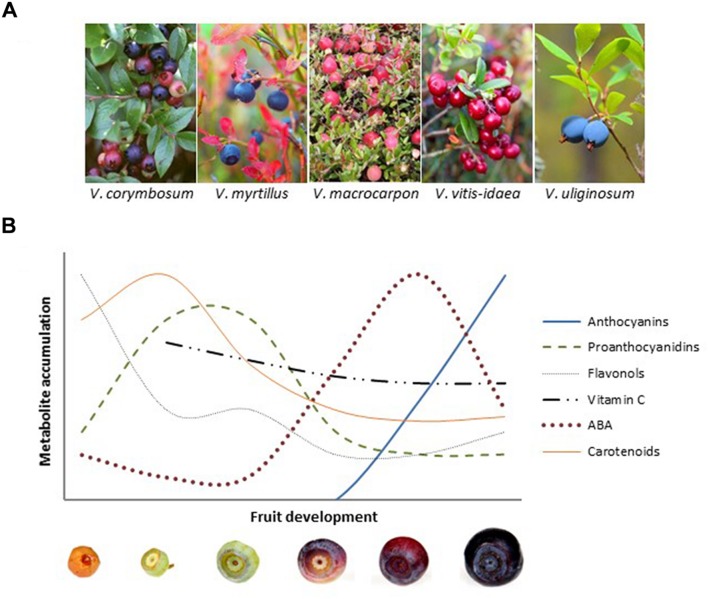
**(A)** *Vaccinium* berries: highbush blueberry (*V. corymbosum*), bilberry (*V. myrtillus*), cranberry (*V. macrocarpon*), lingonberry (*V. vitis-idaea*), and bog bilberry (*V. uliginosum*). **(B)** Schematic representation of the accumulation of key metabolites during bilberry fruit development and ripening. The highest mean values of different compounds are 3960 μg g^-1^ FW for anthocyanins, 216 μg g^-1^ FW for proanthocyanidins, 130 μg g^-1^ FW for flavonols, 82.5 μg g^-1^ FW for vitamin C, 6.2 μg g^-1^ FW for ABA and 81.8 μg g^-1^ DW (14.4 μg g^-1^ FW) for carotenoids, according to Jaakola et al. (2002), Cocetta et al. (2012), and Karppinen et al. (2013, 2016).

Genus *Vaccinium* includes over 450 deciduous or evergreen species distributed in cool temperate regions and mountains of the northern and southern hemispheres. The genus contains economically important cultivated and wild berry species, such as blueberries (e.g., *Vaccinium corymbosum*, *V. angustifolium*), bilberry (*V. myrtillus*), cranberries (*V. macrocarpon*, *V. oxycoccos*), and lingonberry (*V. vitis-idaea*; **Figure 1A**). Numerous studies have given evidence on the beneficial health effects of these berries, for instance in reducing risk of metabolic syndrome and various microbial and degenerative diseases (Kolehmainen et al., 2012; Blumberg et al., 2013; Norberto et al., 2013; Patel, 2014). These health-benefits are mostly attributed to the various phenolic compounds. *Vaccinium* berries are rich with flavonoids, including anthocyanins, flavonols, and proanthocyanidins (Määttä-Riihinen et al., 2004; Rodrigues-Mateos et al., 2012; Ancillotti et al., 2016), which are linked to many biological activities such as anti-inflammatory, antimutagenic, antimicrobial, anticancer, antiobesity, and antioxidant properties (Szajdek and Borowska, 2008; He and Giusti, 2010; Nile and Park, 2014). However, these berries also contain other valuable compounds, such as carotenoids and their derivatives, other flavor compounds and vitamins. This review covers the current knowledge on the developmental and environmental regulation of the biosynthesis of key metabolites in *Vaccinium* berries. Most studies in this topic have been performed on flavonoids but other compounds, such as other phenylpropanoids, carotenoid derivatives, and vitamin C are also covered.

## Developmental Regulation

Development and ripening of fleshy fruits include major changes in fruit structure and in overall metabolism. At the metabolic level, development of *Vaccinium* berries is characterized by the production of high amounts of flavonoids, especially red/blue-pigmented anthocyanins coloring the ripe fruits (**Figure 1A**). At the early stages of berry development, proanthocyanidins, flavonols, and hydroxinnamic acids are the major phenolic compounds in these berries, and the accumulation of anthocyanins begins at the onset of ripening (Jaakola et al., 2002; Vvedenskaya and Vorsa, 2004; Castrejón et al., 2008; Zifkin et al., 2012; Gibson et al., 2013; **Figure 1B**). However, the flavonoid profiles vary between *Vaccinium* berries most of which accumulate anthocyanins only in the skin at ripening. Bilberry, which is recognized as one of the richest source of anthocyanins, accumulates these compounds also in flesh of ripe fruits with 15 different major anthocyanin glycosides identified (Jaakola et al., 2002; Zoratti et al., 2014b). The profile of anthocyanins in ripe bilberries and blueberries comprises glycosides of cyanidin, delphinidin, peonidin, petunidin, and malvidin anthocyanidins (Lohachoompol et al., 2008; Zoratti et al., 2014b). In red-colored *Vaccinium* berries, the profile of anthocyanins is less diverse, cyanidin glycosides being the major anthocyanins in ripe lingonberries, in addition to peonidins in ripe cranberries (Lee and Finn, 2012; Grace et al., 2014; Česonienė et al., 2015). However, proanthocyanidin content in ripe berries is typically higher in red-colored *Vaccinium* berries compared with blueberries. The proanthocyanidin profile of ripe *Vaccinium* berries includes procyanidins with rare A-type linkages (Määttä-Riihinen et al., 2005; Lätti et al., 2011; Grace et al., 2014). In addition to the role of anthocyanins in seed dispersal, the variation in flavonoid profile during berry development is considered to be related in defense responses. For instance, the astringent proanthocyanidins are suggested to provide protection against predation in unripe berries (Harborne, 1997).

Fleshy fruits are traditionally defined as either climacteric or non-climacteric according to the differences in respiration rate and production of ethylene at ripening (Gapper et al., 2013; McAtee et al., 2013; Osorio et al., 2013). In recent years, regulatory role of abscisic acid (ABA) has been established at molecular level in ripening initiation as well as in control of ripening-related anthocyanin biosynthesis of non-climacteric fruits (Jia et al., 2011; Li et al., 2011; Shen et al., 2014; Kadomura-Ishikawa et al., 2015), which includes *Vaccinium* berries. The increase in ABA levels at fruit ripening has been demonstrated in several non-climacteric fruits (Wheeler et al., 2009; Jia et al., 2011; Luo et al., 2014), also in bilberry (Karppinen et al., 2013; **Figure 1B**) and highbush blueberry (Zifkin et al., 2012), suggesting a role for ABA in ripening regulation in *Vaccinium* berries.

The flavonoid biosynthetic routes in plants are well understood and they are known to be regulated mainly through transcriptional control of structural genes (Hichri et al., 2011). The flavonoid pathway has been intensively studied also in *Vaccinium* berries, especially in bilberries and blueberries. The main structural genes have been isolated from bilberry (Jaakola et al., 2002), highbush blueberry (Zifkin et al., 2012), cranberry (Polashock et al., 2002; Sun et al., 2015), and bog bilberry (*V. uliginosum*; Primetta et al., 2015). The studies have indicated the increase in transcription levels of especially chalcone synthase (*CHS*), dihydroflavonol 4-reductase (*DFR*), anthocyanidin synthase (*ANS*), and UDP-glucose flavonoid 3-*O*-glucosyltransferase (*UFGT*) at the ripening stage leading to anthocyanin accumulation.

The key regulators of the flavonoid pathway have been characterized as R2R3 MYB transcription factors, MYC-like basic helix-loop-helix (bHLH) and WD40-repeat proteins, which comprise so called MBW-complex (Ferreyra et al., 2012; Xu et al., 2015). In *Vaccinium* species, potential R2R3 MYB genes involved in flavonoid biosynthesis have been identified in bilberry (Jaakola et al., 2010), highbush blueberry (Li X. et al., 2012; Zifkin et al., 2012; Gupta et al., 2015), and bog bilberry (Primetta et al., 2015). However, the upstream signaling network behind flavonoid biosynthesis is still unclear. At least part of the regulatory network controlling fleshy fruit ripening seems to be conserved during the evolution throughout climacteric and non-climacteric fruits (Seymour et al., 2013). In bilberry, a link between anthocyanin biosynthesis and one of the key regulators of fruit development, a SQUAMOSA-class MADS-box transcription factor, has been demonstrated (Jaakola et al., 2010). However, there are indications that the regulation of anthocyanin biosynthesis might differ in genus *Vaccinium* compared with other species studied so far. In a recent study, white berry mutants of bog bilberry and bilberry deficient in anthocyanins were demonstrated to have a down-regulated MYBPA1-type transcription factor (Primetta et al., 2015), which has been indicated as the key regulator of proanthocyanidin biosynthesis in other fruit species. During recent years, several transcriptome and genome databases of *Vaccinium* berries have been published (Li X. et al., 2012; Rowland et al., 2012; Zifkin et al., 2012; Polashock et al., 2014; Gupta et al., 2015; Sun et al., 2015). From these databases, different families of transcription factors with potential roles in flavonoid biosynthesis have been identified. The databases will serve as an important tool in revealing signaling network involved in regulation of flavonoid biosynthesis and other metabolites in *Vaccinium* species.

Due to the high accumulation of anthocyanins in skin at ripening, carotenoids do not serve as the main pigments attracting seed dispersers in *Vaccinium* berries. However, among fruits *Vaccinium* berries can be considered as good sources of carotenoids, especially lutein and β-carotene (Marinova and Ribarova, 2007; Bunea et al., 2012; Lashmanova et al., 2012; Karppinen et al., 2016). Our recent study on carotenoid biosynthesis has shown that carotenoid content in bilberry fruit is modified during berry development with decreasing trend from small green berry toward ripening berries (Karppinen et al., 2016; **Figure 1B**). This trend is likely to reflect the variable roles of carotenoids during berry development and ripening. In unripe fruits, carotenoids are primarily involved in photosynthesis, whereas during ripening the carotenoid metabolism can turn toward enzymatic degradation to produce apocarotenoids, such as ABA and flavor compounds (McQuinn et al., 2015). Based on study in bilberry, transcriptional regulation of the both key biosynthetic and cleavage genes plays a role in the determination of carotenoid content during berry development and ripening (Karppinen et al., 2016). This indicates coordinately regulated interplay with ABA and carotenoid biosynthetic routes and, furthermore, anthocyanin biosynthesis at bilberry ripening.

Many berries accumulate carotenoid derived volatile flavor compounds at ripening (Beekwilder et al., 2008; García-Limones et al., 2008). However, reports concerning the regulation of formation of these compounds during development and ripening of *Vaccinium* berries are still scant (Rohloff et al., 2009; Gilbert et al., 2013). The aroma of ripe fruits is a complex combination of various flavor compounds, sugars and acids, and variations in these can be high even between the cultivars of the same species (El Hadi et al., 2013). Cultivar-specific differences in volatile profiles have been reported among *Vaccinium* species and highbush blueberry cultivars (Hirvi and Honkanen, 1983; Baloga et al., 1995; Horvat et al., 1996; Forney et al., 2012). The most critical volatiles for the blueberry aroma are considered to be linalool, *trans*-2-hexenol, *trans*-2-hexenal, hexanal, and 1-penten-3-ol, which show increasing trend in highbush blueberries toward fruit maturity (Du et al., 2011; Gilbert et al., 2013).

Fruits and berries are recognized as dietary sources of vitamins. Among berries, *Vaccinium* species have shown to be low or moderate sources of vitamin C with the levels of 0.1–27 mg 100 g^-1^ FW (Bushway et al., 1983; Klein, 2005; Walker et al., 2006; Brown et al., 2012). In bilberry, the levels of vitamin C have shown to be relatively stable during the berry development and ripening (Cocetta et al., 2012; **Figure 1B**), whereas more decrease during berry development was detected in highbush blueberry cultivars (Liu et al., 2015). Moreover, low to moderate levels of other vitamins are reported in *Vaccinium* fruits (Mazza, 2005; Chun et al., 2006). So far, studies on the upstream regulation of vitamin C biosynthesis during berry development in *Vaccinium* spp. species are lacking.

## Environmental Regulation

Environmental factors have a substantial role in the regulation of secondary metabolism in fruits. In general, genetic background determines the secondary metabolite profile of species, whereas environmental factors can cause prominent qualitative and quantitative changes to the metabolite composition. In addition to temperature and light conditions, nutritional status, water balance, diseases and other stresses have been shown to affect the production of secondary metabolites in fruits and berries (Ferrandino and Lovisolo, 2014; Zoratti et al., 2014a; Koshita, 2015). The environmental effects on berry secondary metabolism have been studied widely also in genus *Vaccinium* (**Table 1**). Many studies have focused on the influence of growth conditions on the content of anthocyanins and other phenolic compounds in berries of both wild and cultivated species.

**Table 1:** Main responses of secondary metabolites to environmental effects in *Vaccinium* berries.

Species	Metabolite	Experimental condition	Response	Reference
*Vaccinium corymbosum* (highbush blueberry)	Phenolic compounds	Year/season	Affects significantly the accumulation of total phenolic content and anthocyanins in different cultivars.	Connor et al., 2002b
		Location	Affects significantly the accumulation of total phenolic content and anthocyanins in different cultivars.	Prior et al., 1998; Connor et al., 2002b; Spinardi et al., 2009; Jovančević et al., 2011; Može et al., 2011; Zoratti et al., 2015a
		Light	Anthocyanin accumulation is dependent from high solar radiation.	Zoratti et al., 2015b
		Temperature	The accumulation of anthocyanins is favored at 25°C compared to 30°C. Temperatures lower than 25°C retard ripening and anthocyanin accumulation.	Zoratti et al., 2015a,b
		Post-harvest UV light	UV-B and UV-C increase accumulation of anthocyanins, flavonols, and phenolic acids.	Perkins-Veazie et al., 2008; Wang et al., 2009; Eichholz et al., 2011; Nguyen et al., 2014
	Volatile compounds	Year/season	1-Hexenol, E2-hexanal, and hexanoic acid are the most variable compounds in six cultivars.	Gilbert et al., 2015
		Location	Significant effect on volatile accumulation depending on the cultivar.	Du et al., 2011; Gilbert et al., 2015
		Post-harvest UV light	In cv. Bluecrop, UV-B increases the accumulation of terpenes, ketones, and aldehydes after 2 h of high irradiance whereas alcoholic compounds increased after 24 h.	Eichholz et al., 2011
		Post-harvest visible light	In cv. Scintilla, hexanal and *trans*-2-hexenal are increased after 8 h treatment under red and far-red light compared to white light.	Colquhoun et al., 2013
*V. myrtillus* (bilberry)	Phenolic compounds	Year/season	Affects significantly anthocyanins in bilberry individuals grown in the same location.	Åkerström et al., 2010; Zoratti et al., 2015a
		Location	The accumulation of anthocyanins increases progressively with increasing latitude and altitude.	Lätti et al., 2008; Rieger et al., 2008; Åkerström et al., 2010; Zoratti et al., 2015a,b
		Light	High light increases content of anthocyanins, flavonols, hydroxycinnamic acids, and total phenolics. Blue, red, and far-red light increase the accumulation of anthocyanins and flavonols under controlled temperature conditions.	Jovančević et al., 2011; Zoratti et al., 2014b; Mikulic-Petkovsek et al., 2015
		Photoperiod	Photoperiod of 24 h increases the accumulation of phenolic compounds compared to 12 h day/night.	Uleberg et al., 2012
		Temperature	Higher levels of flavonols and hydroxycinnamic acids in 12°C vs. 18°C. Lower temperatures (10–15°C) favor the accumulation of delphinidins.	Uleberg et al., 2012; Zoratti et al., 2015a,b
*V. macrocarpon* (cranberry)	Phenolic compounds	Light	Visible light increases accumulation of anthocyanins. The highest increase was observed under red light wavelengths.	Zhou and Singh, 2002
		Post-harvest visible light	Increases accumulation of anthocyanins.	Zhou and Singh, 2004

Light conditions have a significant role in the flavonoid metabolism in fruits (Zoratti et al., 2014a), including *Vaccinium* berries, in which especially content and composition of anthocyanins is affected. However, the effect of light on the accumulation of flavonoids in *Vaccinium* berries seems to be regulated in a species-specific manner. Many of the wild *Vaccinium* berries, such as bilberry and lingonberry, grow in shaded habitats and do not require high light for induction of anthocyanin biosynthesis. In these berries, light conditions appear to have merely fine-tuning effects on flavonoid biosynthesis. Recently, it was reported that bilberries grown in sites with higher photosynthetic active radiation contained higher levels of anthocyanins, flavonols, hydroxycinnamic acids, and total phenolics (Mikulic-Petkovsek et al., 2015). The positive effect of light on total phenolics and anthocyanin was also apparent in bilberries grown under sunlight versus shadowed habitats in Montenegro (Jovančević et al., 2011). Although blueberries are also shade-adapted species they seem to require higher solar exposure for normal ripening and anthocyanin accumulation (Zoratti et al., 2015b). In a postharvest study, light had also positive effect on the accumulation of anthocyanins in cranberries (Zhou and Singh, 2004).

In addition to intensity, light effect can be transmitted through perception of other attributes, such as light quality and day length (Zoratti et al., 2014a). Longer days seem to be associated with more intense flavonoid production than shorter days (Jaakola and Hohtola, 2010; Mazur et al., 2014). In bilberry, the effect of photoperiod appears to be one reason for more rapid accumulation and higher concentrations of anthocyanins at northern latitudes compared to southern growth conditions (Uleberg et al., 2012; **Table 1**).

Higher plants utilize multiple photoreceptors to detect different wavelengths of light from ultraviolet (UV)-B to far-red (Möglich et al., 2010; Casal, 2013). In a recent study, a short exposure to specific portions of light spectrum during the early development of bilberry fruit affected the final flavonoid profile in ripe berry (Zoratti et al., 2014b). Especially blue wavelengths increased the accumulation of more hydroxylated anthocyanins; delphinidins, petunidins and malvidins, but not cyanidins and peonidins. Earlier, short treatments with red wavelengths increased anthocyanin accumulation in cranberries compared to white light- or dark-treated berries (Zhou and Singh, 2002). Postharvest studies with UV-B and UV-C light induced anthocyanin accumulation in blueberries (Perkins-Veazie et al., 2008; Wang et al., 2009; Nguyen et al., 2014). However, the signaling pathway from different photoreceptors to flavonoid accumulation and induction of R2R3 MYB transcription factors is not well understood. It is generally accepted that CONSTITUTIVE PHOTOMORPHOGENIC 1 (COP1) acts as a major center of light signaling directly interacting with photoreceptors (Jang et al., 2010; Galvão and Fankhauser, 2015). The MdCOP1 was shown to interact with MdMYB1, a positive regulator of anthocyanin biosynthesis, in apple (Li Y.Y. et al., 2012). A recent study in non-climacteric strawberry fruit revealed that light regulates anthocyanin biosynthesis and related R2R3 MYB transcription factors independently from ABA (Kadomura-Ishikawa et al., 2015). In accordance, additive effect on anthocyanin accumulation was observed under combined light and ABA treatments.

Temperature also affects the composition of secondary metabolites in fruits. In general, cooler temperatures favor biosynthesis of phenolic compounds and vitamin C (Lee and Kader, 2000; Koshita, 2015), whereas both lower and higher temperatures have been shown to decrease the carotenoid biosynthesis in tomatoes and other carotenoid accumulating fruits (Gross, 1991). In *Vaccinium* berries, the temperature effect has been most intensively studied in regards to formation of phenolic compounds. Many studies have concerned the optimal postharvest storage temperature for the stability of phenolic compounds in blueberries and cranberries (Wang and Stretch, 2001; Connor et al., 2002a; Schotsmans et al., 2007). Moreover, Uleberg et al. (2012) showed in a controlled experiment that bilberries produced higher levels of flavonols and hydroxycinnamic acids in 12°C than in 18°C, whereas contents of all anthocyanins, except delphinidin glycosides, were higher in 18°C. Zoratti et al. (2015b) compared the effect of light-temperature combinations contemporary on bilberry and highbush blueberry (cv. Brigitta Blue). For both species, lower temperatures favored the accumulation of anthocyanins in berries. In bilberry, decrease in temperature from 25 to 10°C increased the more hydroxylated forms of anthocyanins in ripening fruits. Similarly, a higher accumulation of anthocyanins was detected in blueberries ripened at 25°C compared to 30°C. However, temperatures below 25°C delayed the ripening of blueberries leading to a slight decrease in all anthocyanins (Zoratti et al., 2015a,b).

Genotype × environment (G×E) interaction related with the formation of secondary metabolites has been studied in many *Vaccinium* species. Connor et al. (2002b) reported significant variation in anthocyanin content among highbush blueberry cultivars across different locations in US, as well as within years in each location indicating a considerable G×E interaction in regulation of anthocyanin content. The G×E interaction was observed also in bilberries affecting especially to accumulation of anthocyanins in relation to differences in latitude and altitude, in which the variation of climatic factors such as temperature, day length, and spectral composition of sunlight are closely correlated (Zoratti et al., 2015a,b). Especially latitude has been shown to influence the accumulation of anthocyanins in *Vaccinium* berries, as a clear increasing trend in anthocyanin content toward north has been reported for North European populations of both bilberry and bog bilberry (Lätti et al., 2008, 2010; Åkerström et al., 2010). Bilberries of the northernmost clones contained not only higher yields of anthocyanins but also a higher proportion of delphinidins whereas more cyanidins accumulated in the berries grown in southern latitudes.

In *Vaccinium* berries, only few studies on the production of secondary metabolites have specifically focused on the effect of increasing altitudes, which are characterized by progressive decrease in temperature and increase in the intensity of visible light. In Northern Italy, higher levels of anthocyanins and ascorbic acid were found in blueberries grown at 600 m a.s.l. compared with 450 m a.s.l. (Spinardi et al., 2009). The same trend in anthocyanin accumulation in bilberries and blueberries was detected along an altitudinal gradient in the Alps of Italy (Zoratti et al., 2015a) as well as in accumulation of anthocyanins and total phenolics in bilberries grown in different altitudes in Montenegro (Jovančević et al., 2011). In the study of Zoratti et al. (2015a), six natural bilberry populations between 1166 and 1829 m a.s.l. showed a clear positive trend in anthocyanin accumulation with increasing elevation, in a 2-year study. In the same study, highbush blueberries showed variation in the anthocyanin accumulation in relation to growth location at different altitude levels, although it resulted to be mostly dependent on the season and particularly temperature. Seasonal differences might explain the results of a 2-year study in Austria (Rieger et al., 2008), where decreasing bilberry anthocyanin contents were found along with increasing altitude (from 800 to 1500 m a.s.l.).

Moreover, environmental factors affect other metabolites in *Vaccinium* berries. In blueberry, G×E interaction was detected in the accumulation of volatile compounds of blueberry aroma profile. Eichholz et al. (2011) and Colquhoun et al. (2013) reported that the accumulation of volatile compounds is affected by light quality, especially UV and red/far-red wavelengths (**Table 1**). The variation of triterpenoid compounds has been studied in bilberry and lingonberry (Szakiel et al., 2012a,b). In lingonberry, dependence of the metabolite levels on geographical origin was detected and considered to be related to length of the growing season and thickness of snow cover.

## Future Prospects

*Vaccinium* berries are among economically the most important fleshy berry fruits worldwide, and the interest in utilization of both cultivated and wild berries of the genus has been showing an increasing trend. The studies reviewed here show that environmental factors can modify the content and composition of secondary metabolites in *Vaccinium* berries, which is important to consider when using these berries in industrial applications. The recent and upcoming data from transcriptome and genome databases along with more accurate tools for metabolite and metabolomics analyses are opening a new era in studies concerning regulation of secondary metabolism in *Vaccinium* species. New methods allow more in depth studies at species and cultivar level and they will increase our understanding on the role of complicated G×E interactions in the regulation of formation of the health-beneficial secondary compounds.

## Author Contributions

All authors (KK, LZ, NN, HH, and LJ) have participated in preparation of the manuscript and have accepted the final version of the manuscript.

## Conflict of Interest Statement

The authors declare that the research was conducted in the absence of any commercial or financial relationships that could be construed as a potential conflict of interest.
